# The Profile of MicroRNA Expression and Potential Role in the Regulation of Drug-Resistant Genes in Cisplatin- and Paclitaxel-Resistant Ovarian Cancer Cell Lines

**DOI:** 10.3390/ijms23010526

**Published:** 2022-01-04

**Authors:** Dominika Kazmierczak, Karol Jopek, Karolina Sterzynska, Michal Nowicki, Marcin Rucinski, Radoslaw Januchowski

**Affiliations:** 1 Department of Histology and Embryology, Poznan University of Medical Sciences, 61-781 Poznan, Poland; karoljopek@ump.edu.pl (K.J.); k.olejniczak@ump.edu.pl (K.S.); mnowicki@ump.edu.pl (M.N.); marcinruc@ump.edu.pl (M.R.); 2 Department of Anatomy and Histology, Collegium Medicum, University of Zielona Gora, Zyty 28 St., 65-046 Zielona Gora, Poland; r.januchowski@cm.uz.zgora.pl

**Keywords:** ovarian cancer, first line chemotherapy, microRNA, drug-resistant genes

## Abstract

Ovarian cancer is the most lethal gynecological malignancy. The high mortality results from late diagnosis and the development of drug resistance. Drug resistance results from changes in the expression of different drug-resistance genes that may be regulated miRNA. The main aim of our study was to detect changes in miRNA expression levels in two cisplatin (CIS) and two paclitaxel (PAC)—resistant variants of the A2780 drug-sensitive ovarian cancer cell line—by miRNA microarray. The next goal was to identify miRNAs responsible for the regulation of drug-resistance genes. We observed changes in the expression of 46 miRNA that may be related to drug resistance. The overexpression of miR-125b-5p, miR-99a-5p, miR-296-3p, and miR-887-3p and downregulation of miR-218-5p, miR-221-3p, and miR-222-3p was observed in both CIS-resistant cell lines. In both PAC-resistant cell lines, we observed the upregulation of miR-221-3p, miR-222-3p, and miR-4485, and decreased expression of miR-551b-3p, miR-551b-5p, and miR-218-5p. Analysis of targets suggest that expression of important drug-resistant genes like protein Tyrosine Phosphatase Receptor Type K (*PTPRK*), receptor tyrosine kinase—*EPHA7*, Semaphorin 3A (*SEMA3A*), or the ATP-binding cassette subfamily B member 1 gene (*ABCB1*) can be regulated by miRNA.

## 1. Introduction

Epithelial ovarian cancer (EOC) is one of the more aggressive gynecological malignancies and is the fifth leading cause of cancer-related deaths in women [1]. According to GLOBOCAN, 295,414 new cases of ovarian cancer and 184,799 of ovarian cancer-related mortality were noted in 2018 [2]. High mortality is related to low diagnosis and development of drug resistance during treatment [3]. Only about 20% of ovarian cancer patients are primarily resistant to chemotherapy, and nearly 80% respond to first-line chemotherapy. However, among these primarily sensitive patients, about 80% will develop drug resistance during treatment [3,4]. This makes the OC an excellent model to study drug resistance development. 

The first-line chemotherapy in OC is always composed of platinum compounds (Cisplatin (CIS) or Carboplatin and taxanes (Paclitaxel (PAC)) [5]. CIS is one of the most important drugs used in chemotherapy of many cancers including testis, lung, and ovarian cancer. The mechanism of action of CIS is based on leading to the formation of intrastrand and interstrand DNA cross-linking [6]. It inhibits DNA replication, RNA transcription, and eventually leads to cancer cell death [6,7]. Cancer cells acquire different mechanisms of CIS resistance. The most important is the repair of damaged DNA via DNA repair systems, expression of drug transporters from the ABC family, such as ABCC2 (MRP2), leading to increased drug reflux and decreased expression of membrane copper or organic anion transporters resulting in reduced drug uptake [6,7,8]. Inside the cell, CIS can be inactivated by sulfhydryl-containing molecules such as glutathione [7,9] and metallothioneins [10].

The second cytostatic drug used in the first-line OC chemotherapy is PAC. The primary mechanism of PAC action is binding to β-tubulin subunits resulting in microtubules stabilization and block of mitosis. It results in cell-division blocking and consequently in cell apoptosis [11]. The most significant in PAC resistance is the overexpression of drug transporters from the ABC family. The most important is glycoprotein P (P-gp, ABCB1), encoded by the *MDR1* gene [12]. 

All these mechanisms of resistance are related to single cancer cells. However, an increasing amount of evidence suggests the role of tumor tissue and interaction between cancer cells and extracellular matrix (ECM) in the development of drug resistance in ovarian cancer [13]. Dense cellular structure and abundant expression of ECM can limit drug penetration into tumor tissue [14]. Some drugs, such as PAC, can bind to cellular macromolecules and limit their availability to cancer cells [15]. Cancer cells interact with ECM molecules, especially colognes by cell surface receptors, that result in activation of intracellular signaling pathways and drug resistance. This mechanism is designated as a cell adhesion-mediated drug resistance (CAM-DR) [16,17,18,19]. 

Although we know many proven and suspected genes involved in drug resistance, mechanisms of their regulation are challenging to get to know in depth. The gene regulation at the post-transcriptional level can be mediated by small non-coding RNA, described as a microRNA (miRNA). Small non-coding RNAs were discovered in the *Caenorhabditis elegans* by the Victor Ambros group in 1993 [20]. The miRNA is a large group of short (19–29 nucleotides), single-stranded RNAs that affect the expression of a vast portion of genes on the post-transcriptional level in organisms belonging to all kingdoms of living [21]. The interaction mechanism between miRNA and mRNA is based on nucleotide complementarity between the seed sequence (6–8 nucleotides) situated in 5′ region of the miRNA and 3′UTR (untranslated region) of mRNA. The complementarity degree is crucial for the type of regulation. The complete or almost complete complementarity leads to degradation of mRNA, and incomplete match results in the inhibited translation [22]. The miRNA regulatory system is pleiotropic. The individual miRNA may affect a lot of genes responsible for the many mechanisms of cancer, including the development of resistance. In contrast, a single mRNA may be targeted by several miRNAs [23]. 

As miRNAs play a significant role in many cellular homeostasis functions, alterations in miRNAs expression concern a vast number of diseases, including cancer [24]. In cancer cells, miRNAs may act as tumor-suppressors or oncogenes. The suppressor miRNAs inhibit tumor development via negative regulation of oncogenes expression, and their expression is decreased. In contrast, miRNAs considered oncogenic are upregulated in cancer cells and promote tumor development via negative regulation of tumor-suppressor genes [25]. So far, in the EOC, many suppressor miRNAs: miR-145, 200c, miR-192 [26], and oncomirs miR-572, miR-1207, miR-551b [26], were described. miRNAs are isolated from tumor tissues and body fluids, including plasma. Circulating miRNAs profile can reflect tissue expression and enable the diagnosis and prognosis of ovarian cancer [27,28].

The increasing amount of evidence points out that short non-coding RNAs seem an essential player in developing drug resistance in various cancers, including ovarian cancer [29,30]. Much research on profile miRNA in ovarian cancer, both in vitro and in vivo, showed significant alterations in the signature of miRNAs in sensitive cell lines/tissues compared to resistant ones [31]. Our previous study showed alterations in the expression of 40 miRNAs in resistant ovarian cancer cell lines compared with sensitive ones [32]. It has been reported that PAC-resistant OC patients with a better prognosis have decreased miR-663 and miR-622 levels. In contrast, overexpression of miR-647 in PAC-sensitive patients correlated with a good prognosis [33]. In another study, the level of miR-31 was downregulated in OC cell lines resistant to PAC and overexpression of miR-31-sensitized ovarian cancer to PAC in vitro and in vivo [34]. We also observed the downregulation of miR-31 in two PAC-resistant and CIS- and TOP-resistant cell lines [32]. Downregulation of miR-31 was also observed in CIS-resistant tumors and cell lines of gallbladder cancer (GBC) [35]. miR-30a was significantly downregulated in the PAC- and CIS-resistant EOC cells, and overexpression of miR-30a enhanced sensitivity to CIS in sensitive and resistant cell lines [36]. Downregulation of miR-29a/b/c in CIS-resistant ovarian cancer cell lines has been observed, and the inhibition of miR-29a expression confer CIS-resistance in sensitive cell lines [37]. Previously, we observed strong downregulation of miR-29a in the TOP-resistant cell line and upregulation of its target gene—*COL3A1*—responsible for TOP-resistance [32]. In PAC-resistant cell line, we observed a correlation between miR-363 downregulation and upregulation of *MDR1* gene-encoding glycoprotein P (P-gp) [32].

The miRNA microarray seems to be an effective and accurate tool for miRNA profiling and selecting individual miRNAs involved in the resistance mechanism. In the current study, we used miRNA microarrays to analyze changes in miRNAs expression in CIS- (A2780CR1, A2780CR2) and PAC-resistant (A2780PR1, A2780PR2) ovarian cancer cell lines. We observed both up- and downregulation of the miRNA genes in all drug-resistant cell lines. We also identified many target genes that can be responsible for drug resistance in investigated cell lines. 

## 2. Results

### 2.1. Gene Chip Quality Assessment

In the present study, we used standard factors such as signal-to-noise ratio internal hybridization and controlled spike-in-controls to determine the quality of analyzed samples preliminarily. Controlled spike-in-controls were spike_in-control-2, spike_in-control-23, spike_in-control-29, spike_in-control-31, spike_in-control-36. Oligos 2, 23, and 29 are RNA, confirming the poly(A) tailing and the ligation. Oligo 31 (poly(A) RNA) confirmed ligation. Oligo 36 is poly(day) DNA and confirmed ligation and the lack of RNases in the RNA sample.

### 2.2. Gene Expression Evaluation and Gene Expression Lists

The current study determined variations in miRNA transcriptional profiles in two CIS-resistant and two PAC-resistant ovarian cancer cell lines. Obtained results provided new information regarding the significance of miRNA expression changes in the resistance development of these drugs in ovarian cancer. miRNA expression changes in CIS- and PAC-resistant sublines in relation to the drug-sensitive A2780 cell line are summarized in Supplementary Table S1. To determine the differentially expressed miRNA, we applied the following cut-off criteria: expression level changes higher than 5-fold or lower than 0.2-fold (up-/downregulation of more than/less than 5 and −5, respectively) and adjusted *p*-value < 0.05. Comparisons of miRNA expression were performed for the drug-resistant cells versus their drug-sensitive counterparts.

### 2.3. miRNAs Expression in CIS- and PAC-Resistant Cell Lines

**Figure 1:**
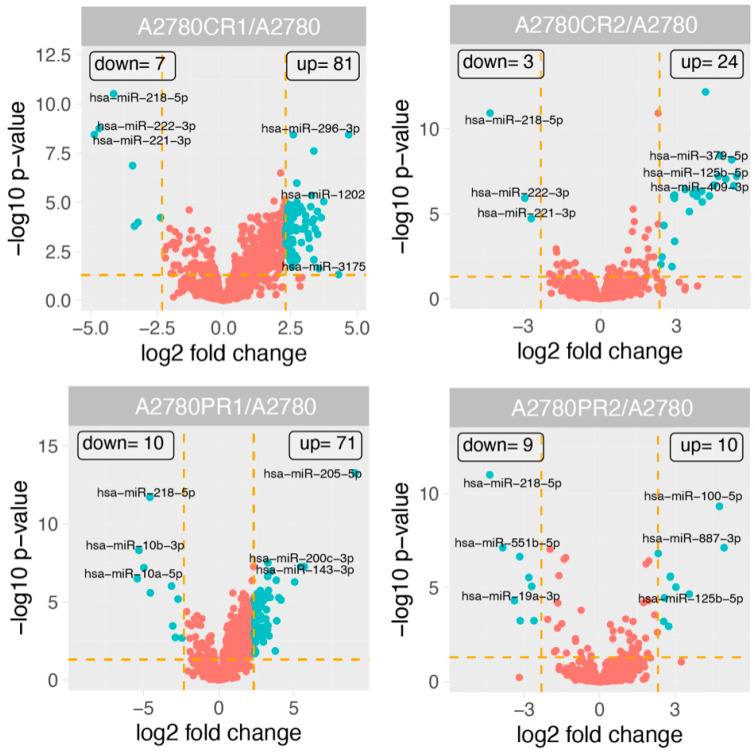
Volcano plots displaying the miRNA with expression levels that were upregulated or downregulated (green dots) by 5-fold or more in drug-resistant cells in relation to the A2780 cells. Red dots indicate miRNAs below cut-off criteria (|fold| > 5, *p* < 0.05).

**Figure 2:**
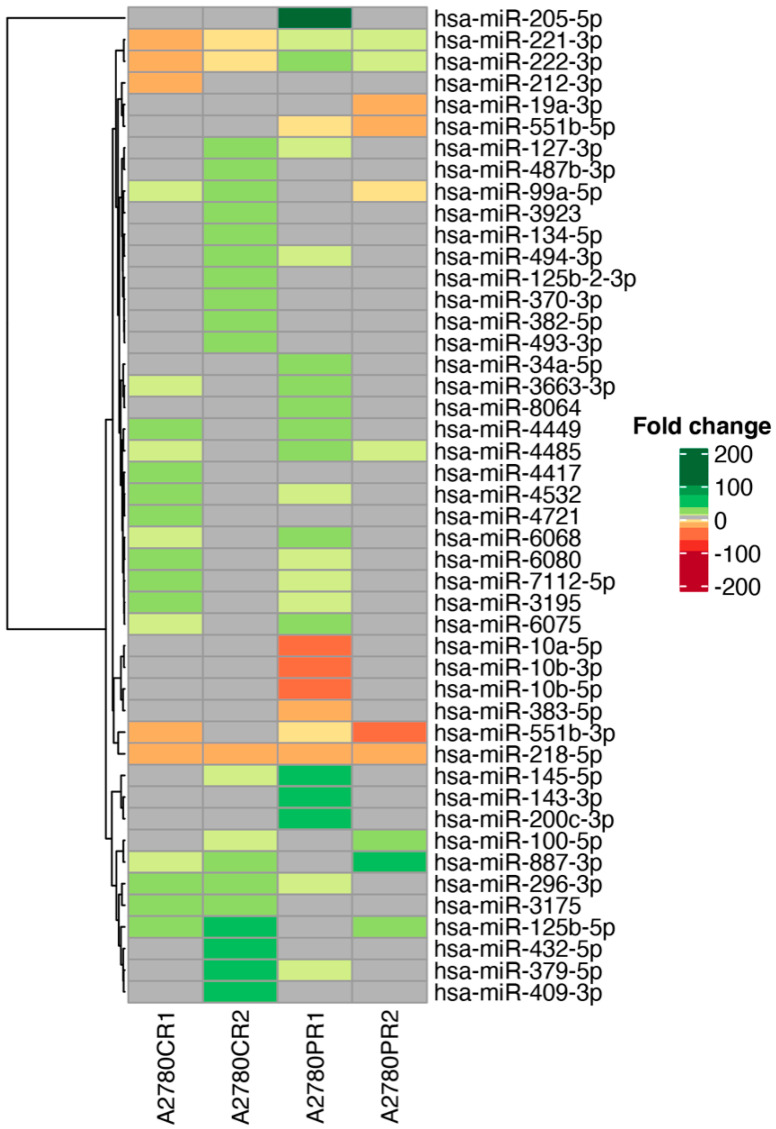
Expression ratios of miRNAs in drug-resistant sublines.

The overall miRNA expression profile of the appropriate experimental groups is shown in Figure 1. We observed changes in the expression of 150 miRNAs (Supplementary Table S1). The expression of 129 miRNAs was elevated in at least one drug-resistant cell line. Expression of 18 miRNAs was downregulated in at least one drug-resistant cell line. The expression of three miRNAs was up- or downregulated, depending on the cell line. To make our analysis more specific for resistance to CIS or PAC for further analysis, we selected only miRNAs that were 1. changed at least five-fold (fold ±5, *p* < 0.05) in two cell lines resistant to the same drug—CIS or PAC, respectively; 2. at least 10-fold changes in miRNA expression in one drug-resistant cell line. As a result, we observed changes in expression of 46 miRNAs (Table 1, Figure 2). The level of expression of 34 miRNAs was upregulated in at least one drug-resistant cell line. Expression of nine miRNAs was downregulated in at least one drug-resistant cell line, while the expression of three miRNAs was up- or downregulated depending on the cell line.

The expression of three miRNAs was changed in all investigated cell lines, and among them, expression of miR-218-5p was downregulated in all investigated cell lines. Expression of miR-221-3p and miR-222-3p was downregulated in both CIS-resistant cell lines but upregulated in both PAC-resistant cell lines. Expression of five miRNAs was changed in three of four cell lines, and among them expression of miR-125b-5p, miR-887-3p was upregulated in both CIS-resistant cell lines and in the A2780PR2 cell line, expression of miR-296-3p was upregulated in both CIS-resistant cell lines and in the A2780PR1 cell line, expression of miR-99a-5p was upregulated in both CIS-resistant cell lines but downregulated in the A2780PR2 cell line, and expression of miR-551b-3p was downregulated in both PAC-resistant cell lines and the A2780CR1 cell line. Additionally, miR-551b-5p was downregulated in both PAC-resistant cell lines and expression of miR-4485 was upregulated in both PAC-resistant cell lines Thus, the upregulation of miR-125b-5p, miR-99a-5p, miR-296-3p, miR-887-3p, and miR-4485, and downregulation of miR-218-5p, miR-221-3p, and miR-222-3p, was specific to CIS-resistance. On the other hand, upregulation of miR-221-3p and miR-222-3p, and downregulation of miR-551b-3p, miR-551b-5p, and miR-218-5p, was specific for PAC-resistance.

Expression of another 37 miRNAs was changed at least 10-fold in one resistant cell line. Among them, expression of 31 miRNAs was upregulated, and expression of 6 miRNAs was downregulated. 

Among all analyzed miRNAs, changes in expression of 19 miRNAs were very significant, at >20 fold. An enormous increase in expression was observed for miR-205-5p, which was upregulated over 541-fold in the A2780PR1 cell line. In this cell line, we also observed 51-fold increase in miR-143-3p expression. Among other strongly upregulated miRNAs, we can distinguish those upregulated over 40-fold (miR-125b-5p in the A2780CR2 cell line, miR-200c-3p in the A2780PR1 cell line, those upregulated over 30-fold (miR-145-5p in the A2780PR1 cell line and miR-379-5p, miR-409-3p, miR-432-5p—all in the A2780CR2 cell line). Four miRNAs were upregulated over 20-fold: miR-100-5p in the A2780PR2 cell line, miR-296-3p in both CIS-resistant cell lines, and miR-127-3p and miR-487b-3p in the A2780CR2 cell line.

Among strongly downregulated miRNAs, we observed strong downregulation: over 40-fold of miRNA-10a-5p in the A2780PR1 cell line and miR-551b-3p in the A2780PR2 cell line. In addition, strong downregulation, over 30-fold, of miR-10b-5p and miR-10b-3p was observed in the A2780PR1 cell line. Other miRNAs were downregulated over 20-fold: miR-218-5p in both PAC-resistant cell lines, miR-221-3p and miR-222-3p in the A2780CR1 cell line and miR-383-5p in the A2780PR1 cell line.

### 2.4. Analysis of Target Genes Expression

We further examined whether differentially expressed miRNAs are involved in the regulation of genes responsible for the drug resistance development. Based on the assumption that an increase in miRNA expression causes a decrease of target gene expression and vice versa, for further analysis, we selected only those target genes for which the fold change value was inversely correlated with miRNA fold change. We applied the same cut-off values for both miRNAs and their targets—at least 5-fold up/down and adjusted *p* value <0.05. Target expression below cut-off criteria was considered as “not significant (NS)” when the target gene lists were constructed. We used our microarray data, which has been published previously [24,25,26]. Using these criteria (changes in expression 5-fold up or down), we found targets for 106 from 150 miRNAs.

For further analysis, target genes related to drug resistance, extracellular matrix, and cancer stem cell biology were selected from the whole population of target genes using the following key words in the Gene Ontology (GO) database: response to a drug, drug transport, extracellular space, extracellular matrix, collagen-containing extracellular matrix, a stem cell. Previously, we described that these genes belong to differentially expressed genes in drug-resistant cell lines [24,25,26]. 

**Figure 3:**
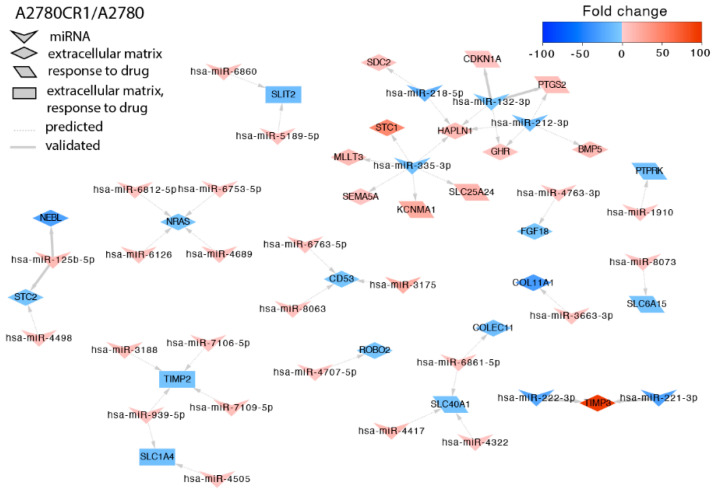
Regulation of selected target genes by miRNAs in the A2780CR1 cell line.

In the A2780CR1 cell line, we identified targets for 28 miRNAs (Figure 3). Among them, the downregulation of miR-221-3p and miR-222-3p correlated with high upregulation of *TIMP3* (*Tissue Inhibitor of Metalloproteinases 3*). Decreased expression of the *ROBO2* (*Roundabout Guidance Receptor 2*) gene correlated with increased expression of miR-4707-5p. We also observed that downregulation of SLC transporters (solute carrier) were regulated by upregulation of miRs. Among them, miR-939-5p and miR-4505 upregulation correlated with *SLC1A4* gene downregulation, upregulation of miR-6881-5p, miR-4322, and miR-4417 correlated with *SLC40A1* downregulation, and upregulation of miR-8073 correlated with downregulation of *SLC6A15*. In contrast, miR-335-3p downregulation correlated with *SCL25A24* upregulation. Decreased expression of *PTPRK* (*Protein Tyrosine Phosphatase Receptor Type K*) correlated with miR-1910 upregulation.

**Figure 4:**
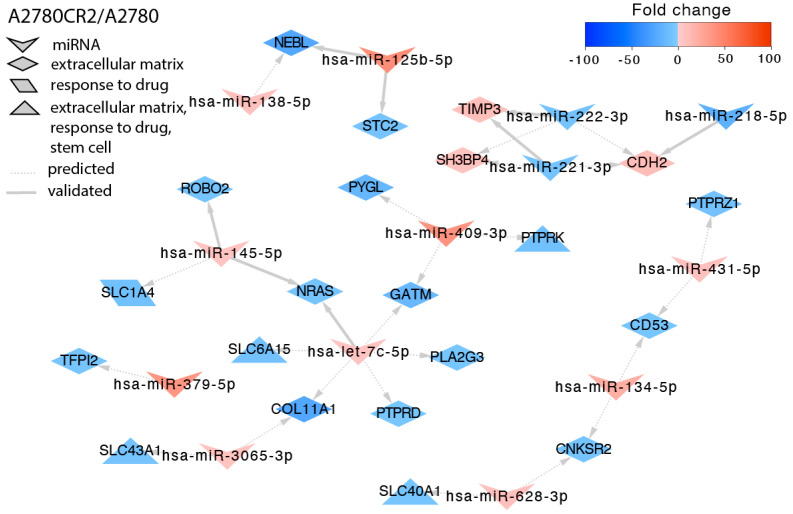
Regulation of selected target genes by miRNAs in the A2780CR2 cell line.

In the second CIS-resistant cell line—A2780CR2—we identified targets for 15 miRNAs (Figure 4). Similar to in the A2780CR1 cell line, upregulation of *TIMP3* resulted from downregulation of miR-221-3p and miR-222-3p. We also observed that increased expression of CDH2 (*Cadherin 2*, *Type 1*, *N-Cadherin*) resulted from downregulation of miR-222-3p. Decreased expression of the *ROBO2* gene inversely correlated with upregulation of miR-145-5p. Similar to previously, we also observed downregulation of SLC transporters. *SLC1A1* downregulation correlated with miR-145-5p upregulation, decreased expression of *SLC43A1* correlated with upregulation of miR-3065-3p, upregulation of let-7c-5p correlated with downregulation of *SLC6A15,* and upregulation of miR-628-3p correlated with downregulation of *SLC40A1*. In this cell line, we also observed downregulation of tyrosine phosphatases. Among them, *PTPRK* was regulated by miR-409-3p, *PTPRD* (*Protein Tyrosine Phosphatase Receptor Type D*) by let-7c-5p, and *PTPRZ1* (*Protein Tyrosine Phosphatase Receptor Type Z1*) by miR-431-5p. 

**Figure 5:**
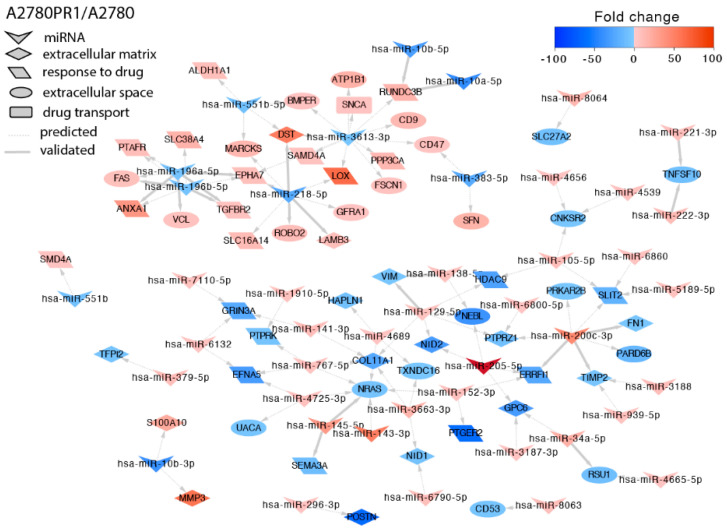
Regulation of selected target genes by miRNAs in the A2780PR1 cell line.

The second drug used in the first line of ovarian cancer chemotherapy is PAC. Thus, we also analyzed miRs expression in two PAC-resistant cell lines. In the A2780PR1 cell line, we identified targets for 39 miRNAs (Figure 5). *LAMB3* overexpression correlated with downregulation of miR-218-5p. In contrast to CIS-resistant cell lines, *ROBO2* overexpression correlated with downregulation of miR-218-5p. We also observed that *TNFSF10* gene downregulation correlated with overexpression of miR-221-3p and miR-222-3p. We also observed regulation of SLC transporters by miRNAs. Among them, miR-218-5p downregulation correlated with *SLC16A14* upregulation, and miR-8064 upregulation correlated with *SLC27A2* downregulation. As increased protein phosphorylation is a key feature of drug-resistant cells, we also observed downregulation of protein phosphatases by miRNAs. *PTPRK* was regulated by miR-1910-5p and miR-767-5p, and *PTPRZ1* was regulated by miR-129-5p, miR-6800-5p, and miR-200c-3p. In contrast, upregulation of *EPHA7* gene-encoding protein kinase (*EPH Homology Kinase 3*) correlated with downregulation of miR-196a-5p, miR-196b-5p, and miR-218-5p. We also observed increased expression of genes involved in cancer stem cells biology. Upregulation of *ALDH1A1* (*Aldehyde Dehydrogenase 1 Family Member A1*) resulted from miR-551b-5p downregulation. LOX (*Lysyl Oxidase*) upregulation correlated with downregulation of miR-218-5p and miR-3613-3p. Additionally, downregulation of miR-3613-3p correlated with expression of other drug-resistant genes: *SAMD4A* (*Sterile Alpha Motif Domain Containing 4A*) and *RUNDC3B* (*RUN Domain-Containing Protein 3B*). Additionally, expression of *SAMD4A* was also regulated by miR-551b, and miR-10a-5p and miR-10b-5p regulated expression of *RUNDC3B*. Decreased expression of *SEMA3A* (*Semaphorin 3A*) resulted from increased expression of miR-145-5p.

**Figure 6:**
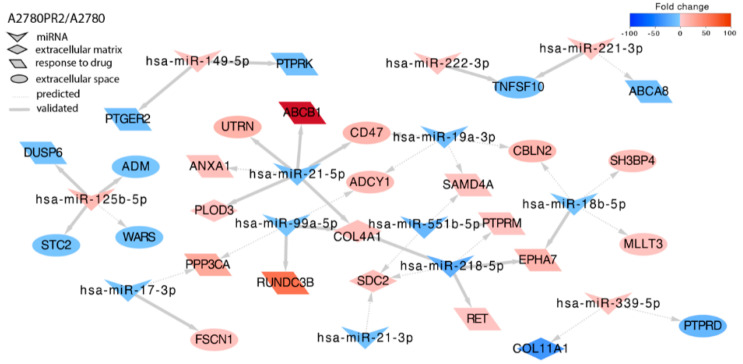
Regulation of selected target genes by miRNAs in the A2780PR2 cell line.

In the second PAC-resistant cell line, we identified targets for 13 miRNAs (Figure 6). Increased expression of *COL4A1* (*Collagen Type IV Alpha 1 Chain*) resulted from downregulation of miR-99a-5p. Similar to in the A2780PR1 cell line, we also observed that *TNFSF10* downregulation correlated with overexpression of miR-221-3p and miR-222-3p. As in other resistant cell lines, we also observed decreased expression of protein phosphatases that correlated with increased expression of specific miRNAs. In detail: *PTPRK* was regulated by miR-149-5p, and *PTPRD* by miR-339-5p. In contrast, protein kinase *EPHA7* was increased and correlated with miR-18b-5p and miR-218-5p downregulation. We also observed increased expression of *SAMD4* associated with decreased expression of miR-19a-3p and miR-551b-5p, and increased expression of *RUNDC3B* associated with miR-99a-5p. The most important protein responsible for PAC-resistance in this cell line is glycoprotein P (P-gp) encoded by the *MDR1*/*ABCB1* (*Multidrug Resistance Protein 1*/ *ATP Binding Cassette Subfamily B Member 1*) gene. We could observe that a strong increase in *ABCB1* expression correlated with decreased expression of miR-21-5p. 

## 3. Discussion

The development of drug resistance is a significant problem in ovarian cancer chemotherapy. Hundreds of genes are known to play a role in this process, and new ones are still being described. Among mammalian genes, up to 60% can be regulated by miRNA [22]. As miRNAs are considered a new therapeutic tool in many diseases, including cancer [38], the knowledge about the regulation of drug-resistance genes by miRNAs seems to be very important from a clinical point of view.

The present results show the correlation between resistance to cytotoxic drugs and the expression of genes encoding microRNAs. Here, we analyzed the expression of miRNA genes in ovarian cancer cell lines resistant to CIS and PAC—drugs used in first- line chemotherapy of OC [5]. In contrast to studies where only one pair of sensitive/resistant cell lines is tested, in this study, changes in miRNA expression in four drug-resistant cell lines derived from one parental cell line were examined. Our results also compared miRNA expression in twin cell lines resistant to CIS and PAC. It is a unique model for such research.

In these cell lines, we previously observed increased expression of genes known from resistance to CIS, such as MRP2 in both CIS-resistant cell lines, or PAC, such as *MDR1*/P-gp in both PAC-resistant cell lines [39,40]. We also observed changes in the expression of many genes encoding ECM molecules [41]. Additionally, we noted up- or downregulation of “new drug-resistant genes” that may be related to CIS and/or PAC resistance, as the changes were observed in three cell lines resistant to the same drug [42,43,44]. Additionally, in drug-resistant cell lines, we observed population of ALDH1A1-positive cancer stem cells (CSCs) [45,46]. However, we did not observe any cross-resistance to PAC in CIS-resistant cell lines, and there was a low level of resistance to CIS in the A2780PR2 cell line [39]. To make our analysis more specific, we started our investigation from miRNA changes in two cell lines resistant to CIS or PAC, respectively. Among 46 miRNAs analyzed in this experiment, the expression of four (miR-125b-5p, miR-99a5p, miR-296-3p, and miR-887-3p) was upregulated and three (miR-218-5p, miR-221-3p, and miR-222-3p) was downregulated in both CIS-resistant cell lines, suggesting their role in CIS resistance. In contrast to our study, the research conducted on colon cancer [47] and gallbladder cancer [48] pointed to downregulation of miR-125b-5p in CIS-resistant models and suggested its suppressor role in resistance to CIS both in vitro and in vivo. The differences between these and our results can result from a different mechanism of CIS resistance in investigated cell lines. Both CIS-resistant cell lines were also characterized by overexpression of miR-99a-5p. The results in line with our research were presented on gastric cancer (GC). Significant upregulation of miR-99a-5p was observed in CIS-resistant GC tissues as well as in CIS-resistant cell lines [49]. An elevated level of miR-99a-5p was also observed in serum from ovarian cancer patients and promoted cancer cells invasion in vitro [50]. Thus, miR-99a-5p upregulation seems to be related to OC invasion and drug resistance. The downregulation of miR-218-5p resulted in increased resistance to CIS in this cancer, as well [51]. In ovarian cancer study, decreased expression of miR-218-5p was observed in ovarian cancer tissue in comparison to normal ovary and in ovarian cancer cell lines in comparison to normal ovarian cell lines. Furthermore, decreased level was observed in patients with FIGO III, IV compared to with FIGO I, II and in patients with the presence of metastasis, suggesting a significant role of miR-218-5p downregulation in ovarian cancer progression. As metastasis are usually more resistant than primary tumors, and we observe reduction of miR-218-5p expression in drug-resistant cell lines, we can suggest that this miR can be associated with both processes [52]. The last two miRs downregulated in both CIS-resistance cell lines were miR-221-3p and miR-222-3p. The relationship between the expression of these miRs and CIS-resistance is not known so far. However, ovarian cancer study has shown that decreased level of both miR-221-3p and miR-222-3p was linked to lower overall survival (OS) of patients [53,54]. Furthermore, more malignant cells showed reduced expression of miR-221-3p [54], which was associated with higher tumor grade (lower in G3 vs. G1, G2) and correlated with tumor growth in vivo [54]. In vitro experiments indicated that miR-222-3p inhibited EOC cell proliferation and migration [54]. In summary, the downregulation of both miRs seems to be related to ovarian cancer progression and CIS-resistance development. Based on the results obtained, we can assume that miR-99a-5p, miR-218-5p, miR-221-3p, and miR-222-3p may play a role in ovarian cancer progression and drug resistance. However, the detail of the role requires functional study.

In contrast to CIS-resistant cell lines in both PAC-resistant cell lines, both miR-221-3p and miR-222-3p were upregulated. We did not find any data concerning miR-222-3p expression in PAC-resistance cancer or cancer cell lines. On the contrary to our results, strong downregulation of miR-221-3p was observed in non-small-cell lung cancer (NSCLC) PAC-resistant cell line (A549/Taxol) compared to parental control [55]. Those differences may be due to other PAC-resistance mechanisms in investigated cell lines. Both strands of miR-551b (miR-551b-3p and miR-551b-5p) had a downregulated expression in both PAC-resistant cell lines. miR-551b-3p, expression level was also significantly downregulated in the gastric cancer tissues, which correlated with the degree of differentiation (i.e., tumor grade), TNM stage, and lymph node metastasis [56]. Downregulation of miR-551b-5p was reported in breast cancer cell lines and tissues and was an indicator of poor overall survival [57]. We observed downregulation of both miRs in PAC-resistant cell lines while in others during tumor progression. As a tumor progression is usually related to higher drug resistance, miR-551b-3p and miR-551b-5p downregulation can be related to both processes. 

Next, we analyzed other miRNAs whose expression was significantly altered in tested drug-resistant cell lines. However, the pattern of their expression differed between cell lines resistant to the same drug. Most highly overexpressed or downregulated miRNA were observed in PAC-resistant cell lines. The highest expression was observed for miR-205-5p in the A2780PR1 cell line (over 500-fold), suggesting its significant role in resistance to PAC. The role of miR-205-5p overexpression in PAC resistance seems to be supported by other studies. miR-205-5p was upregulated in endometrial cancer (EC) tissues and cell lines compared with respective control [58]. Here, we observe increased expression in the A2780 cell line that is isolated from endometrioid ovarian cancer [59]. Hence, the similar pattern of expression can result from similar origin of both cancers. Expression of miR-205-5p was also related to ovarian cancer progression, as plasma level of miR-205-5p was significantly elevated in ovarian cancer patients compared to control and in poorly differentiated tumors compared to well/moderate differentiated ones [60]. Overexpression of miR-205-5p was also elevated in HGSC tumors in comparison to ovarian surface epithelium [61]. Thus, overexpression of miR-205-5p seems to be related to both drug resistance and ovarian cancer progression. The upregulation of miR-200c-3p in PAC-resistant cell lines seems to be following its role in OC progression. The miR-200c-3p level was upregulated in ovarian cancer tissue and serum exosomes and is related to the presence of distinct metastasis [62]. In HGSC patients, high miR-200c-3p expression was associated with poor progression-free and overall survival [61]. In another HGSC study, increased expression of miR-200c-3p in tumor tissue was observed in stage III/IV, compared to stage I/II [63]. Thus, increased expression of miR-200c-3p may be related to ovarian cancer progression and PAC resistance. An elevated level of miR-100-5p in the A2780PR2 cell line seems to be related to PAC resistance. This miR was also observed in PAC-resistant prostate cancer cell lines [64] and ovarian cancer tissue [65]. Downregulation of miR-10a-5p in the A2780PR1 cell line may be related to PAC resistance in ovarian cancer, as we previously also observed downregulation of miR-10a-5p expression in two other PAC-resistant ovarian cancer cell lines [32]. Expression of miR-10a-5p was downregulated in ovarian cancer cell lines, tissue, and serum, and correlated with FIGO stage (lower in III/IV), presence of lymph node metastasis, and poor OS [66]. Therefore, we hypothesize that it may influence ovarian cancer progression and drug resistance. The last downregulated miR analyzed in this study was miR-383-5p. The upregulation of this miR enhanced the sensitivity of OC cell lines to PAC [67]. Thus, this miR’s downregulation should enhance PAC resistance, which we could observe in our study. Summarizing this part of the analysis, we can conclude that changes in the expression of miR-205-5p, miR-200c-3p, miR-100-5p, miR-10a-5p, and miR-383-5p are supported by other results, and we suggest their role in PAC resistance and/or ovarian cancer progression.

Presented results may indicate the potential role of miRNA in drug-resistance development. It concerns especially miRNAs down/upregulated in two cell lines resistant to CIS or PAC. We also observed differences in the expression of particular miRNAs between two cell lines resistant to the same drug. It emphasizes the complexity of the drug-resistance mechanism development, which we could also observe for the protein-encoding genes involved in this process [40,41,42].

Interpretation of miRNA results is much more complicated than of protein-coding genes expression. For example, expression of ABC transporters such as P-gp or BCRP is always related to PAC and DOX or TOP resistance, respectively [12]. miRNA influence is more comprehensive, as one miRNA may regulate the expression of many genes, yet one miRNA can be a target of different miRs. Another issue is that miRNAs do not directly regulate drug-resistance genes expression but modulate the signaling pathways regulating their expression. 

To better understand the role of investigated miRNA in drug resistance, the second part of our investigation was devoted to reverse correlation between the expression of miRs and their target genes. We focused mainly on genes previously described by ours in the context of drug resistance [40,41,42].

Previously, both CIS-resistant cell lines presented the upregulation of *TIMP3* belonging to tissue inhibitors of metalloproteinases gene family, involved in the degradation of the ECM [41]. Here, the upregulation of *TIMP3* correlated with a downregulation of miR-221-3p and miR-222-3p in both CIS-resistant cell lines. Regulation of *TIMP3* by miR-221-3p was described in diabetic retinopathy [68] and by miR-221-3p and miR-222-3p chronic liver disease [69]. Overexpression of miR-222-3p was associated with the downregulation of *TIMP3* in osteosarcoma and promoted cells proliferation and invasion [70]. Thus, the involvement of miR-221-3p and miR-222-2p seems to be a universal mechanism of *TIMP3* gene regulation.

Among other ECM genes regulated by miRNA, we can distinguish *CDH2* in the A2780CR2 cell line, *LAMB3* in the A2780PR1 cell line, and *COL4A1* in the A2780PR2 cell line. *CDH2* upregulation was associated with decreased expression of miR-218-5p. Association between *CDH2* and miR-218-5p has been reported by others [71]. We could observe increased expression of *LAMB3* in PAC-resistant cell lines. Overexpression of LAMB3 was also observed in platinum-resistant ovarian cancer patients [72], suggesting this gene’s role in drug resistance. In our study, increased expression of *LAMB3* correlated with decreased expression of miR-218-5p. This is supported by head and neck squamous cell carcinoma (HNSCC) study, where direct regulation of *LAMB3* by miR-218 has been described and targeting of *LAMB3* by miR-218 resulted in significant inhibition of cell migration and invasion [73]. Upregulation of *COL4A1* in the A2780PR2 cell line correlated with miR-21-5p downregulation. Regulation of *COL4A1* expression by miR-21-5p was described previously in papillary thyroid cancer [74]. ROBO2 is a transmembrane receptor for the Slit homolog 2 protein and functions in axon guidance and cell migration [75]. It is a putative tumor-suppressor gene, and its expression was reduced in different cancers, including ovarian cancer. Decreased expression of ROBO2 was observed in primary cultures of ovarian cancer epithelial cells, compared to normal OSE, and in poorly differentiated SKOV-3 cells, compared to the more differentiated PEO-14 cells [76]. We observed downregulation of *ROBO2* in both CIS-resistant cell lines, suggesting its role in resistance to this drug. Regulation of *ROBO2* by miR-145-5p was also observed in primary neurons [77]. In contrast, *ROBO2* expression was upregulated in the A2780PR1 cell line and was associated with miR-218-5p downregulation. Regulation of *ROBO2* by this miR was also noted in vascularization of the retina, where *ROBO2* was a direct target of miR-218-5p [78]. We could follow a different pattern of *ROBO2* expression in CIS- and PAC-resistant cell lines regulated by miRNA. Kinases and phosphatases activate/deactivate signaling proteins by reversible phosphorylation resulting in up/downregulation signal transduction and changes in genes expression, cellular metabolism, and rate of cell proliferation [79]. The characteristic feature of cancer cells is a high level of protein phosphorylation [80], which plays an important role in the development of drug resistance [81,82]. Changes in protein phosphorylation can be related to increased expression of protein kinases and decreased expression of protein phosphatases. Previously, we observed reduced expression of PTPRK in 17 drug-resistant ovarian cancer cell lines [83]. Here, we observed that in both CIS- and PAC-resistant cell lines, the downregulation of *PTPRK* correlated with upregulation of different miRNAs. In the A2780CR1 and A2780PR1 cell lines, we observed a relationship between *PTPRK* downregulation and upregulation of miR-1910-5p. Additionally, in the A2780PR1 cell line, we observed regulation by miR-767-5p. However, in the A2780CR2 cell line, *PTPRK* was regulated by miR-409-3p, and in the A2780PR2 cell line by miR-149-5p. Thus, expression of the same gene, even in twin cell lines resistant to the same drug, was regulated by different miRNA. Increased signal transduction can also be associated with increased kinases activity and expression. Previously, we described increased expression of EPHA7 (EPH receptor A7) in three PAC-resistant cell lines [42]. *EPHA7* encodes the ephrin receptor, which belongs to the protein-tyrosine kinase family and functions as receptor-tyrosine kinase, resulting in activation of ERK pathway [84]. Its increased expression was observed, among others, in gallbladder adenocarcinoma, where it was an independent poor prognostic marker, and its upregulation was associated with carcinogenesis, disease progression, and a poor prognosis [85]. Previously, in another PAC-resistant cell line, we observed that *EPHA7* overexpression correlates with downregulation of miR-18b [32], which was also supported by other studies [86]. Here, we noticed that *EPHA7* overexpression correlates with miR-18b-5p downregulation in the A2780PR2 cell line. It suggests the role of this miR in *EPHA7* gene regulation. Additionally, in the A2780PR1 cell line we observed regulation of *EPHA7* expression by miR-196a-5p and miR-196b-5p. Regulation of EPHA7 by miR-196b was also observed in esophageal squamous cell carcinoma [87]. Semaphorins are proteins involved in many developmental processes and cancer progression [88]. The downregulation of SEMA3A as a tumor suppressor gene was described in many cancers, including ovarian cancer [89]. Previously, we described downregulation of *SEMA3A* expression in three from four ovarian cancer PAC-resistant cell lines [44], and in the W1-PAC-resistant cell line, its expression was regulated by miR-145 [32]. Here, the downregulation of *SEMA3A* in the A2780PR1 cell line also correlated with miR-145 upregulation. This regulation is also supported by others [90].The most important gene involved in PAC resistance is glycoprotein P, encoded by *MDR1*/*ABCB1* [12]. Its expression was reported by ours previously in all PAC-resistant cell lines [42]. Here, the high upregulation of the *MDR1* gene correlated with the downregulation of miR-21-5p. *MDR1* has also been identified as a target of miR-21 in colorectal adenocarcinoma [91]. In summary, we have identified many miRNA-target pairs, and some of them were described by others. Other ones, we found for the first time. Furthermore, even the tween twin cell lines resistant to the same drug present different levels of regulation, where one target may be regulated by different miRNA. It proves that the mechanism of action relying on miRNA regulation is complex and complicated.

## 4. Materials and Methods

### 4.1. Reagents 

Cisplatin, Doxorubicyn, Topotekan, and Paclitaxel were obtained from Sigma (St. Louis, MO, USA). RPMI-1640 medium, fetal bovine serum, penicillin, streptomycin, amphotericin B (25 μg/mL), and L-glutamine were also purchased from Sigma. QIazol Lysys Reagent, miRNeasy Mini Kit, and RNeasy MinElute Cleanup Kit were obtained from Qiagen (Hilden, Germany). GeneChipTM miRNA 4.1 Array Strip, FlashTagTM Biotin HSR RNA Labeling Kits, GeneAtlasTM Hybridization, Wash, and Stain Kit for miRNA Arrays were obtained from Afymetrix (Santa Clara, CA, USA).

### 4.2. Cell Lines and Cell Culture 

The human ovarian carcinoma A2780 cell line was purchased from ATCC. A2780 sublines that were resistant to CIS (A2780CR1, A2780CR2 (A2780 cisplatin-resistant)), and PAC (A2780PR1 and A2780PR2 (A2780 paclitaxel-resistant)) were obtained by exposing A2780 cells to the relevant drugs at gradually increasing concentrations. The final concentrations used to select the resistant cells were 1000 ng/mL of CIS for both A2780CR1 and A2780CR2 cell lines, 300 ng/mL of PAC for A2780PR1 cell line, and 1100 ng/mL of PAC for A2780PR2 cell line, as described previously [39]. They were twofold greater than the concentrations in the plasma 2 h after intravenous administration [92]. According to parental drug-sensitive cell lines, the increase in resistance was as follows: 4.09-fold for A2780CR1 vs. A2780, 3.29-fold for A2780CR2 vs. A2780, 146-fold for A2780PR1 vs. A2780, and 1202-fold for A2780PR2 vs. A2780, as we described previously [39]. All cell lines were maintained as a monolayer in the complete medium (MEM medium supplemented with 10% (*v*/*v*) fetal bovine serum, 2 pM L-glutamine, penicillin (100 U/mL), streptomycin (100 U/mL) and amphotericin B (25 µg/mL)) at 37 °C in a 5% CO_2_ atmosphere.

### 4.3. miRNA Isolation

miRNA was isolated using a Qiagen reagent kit following the producer’s protocol. The concentration and quality of isolated miRNA was quantified by measuring absorbance at 260 nm and 280 nm. The 260/280 nm ratio was used to estimate the degree of protein contamination. In all samples, the 260/280 nm ratio ranged from 1.8 to 2.0. From each study group, four samples were subjected to miRNA expression profiling by microarray. (N/group = 4).

### 4.4. Microarray Preparation, Hybridization, and Scanning

The profiling of miRNA expression was performed using Affymetrix platform-based microarrays with GeneChip™ miRNA 4.1 Array Strip (Thermo Fisher Scientific, Waltham, MA, USA). The detailed technical procedure is described elsewhere [93,94]. Each microarray was designed following the miRBase Release 20 database, including complementary probes for 2578 human mature miRNA, 1996 human snoRNA, CDBox RNA, H/ACA Box RNA, scaRNA, and 2025 human pre-miRNA. Preparation of miRNA to hybridization step was performed using the FlashTagTM Biotin HSR RNA Labeling Kit (Thermo Fisher Scientific, Waltham, MA, USA). Briefly, 150 ng of miRNA was subjected to the poly(A) tailing and biotin ligation under the producer’s protocol. Biotin-labeled miRNA were then hybridized to GeneChip™ miRNA 4.1 Array Strip (20 h, 48 °C). Microarrays were then washed and stained according to the technical protocol using the Affymetrix GeneAtlas Fluidics Station (Affymetrix, Santa Clara, CA, USA). The array strips were scanned using an Imaging Station of GeneAtlas System (Thermo Fisher Scientific, Waltham, MA, USA).

### 4.5. Microarray Analysis and miRNA Gene Screening

The preliminary analysis of scanned microarrays was carried out using Affymetrix GeneAtlas Operating Software (Affymetrix, Santa Clara, CA, USA). The quality of the miRNA expression profile was verified using default quality control criteria determined by the software. BioConductor libraries from the statistical programming language “R” were used for further analyses. The raw CEL files obtained as a result of microarray scanning were imported into this programming environment. The Robust Multiarray Average (RMA) normalization algorithm (implemented in the Affy library) was used for normalization, background correction, and expression calculation [95]. The biological annotation for human miRNA was retrieved from the pd.mirna 4.1 library, where tabulated biological descriptors were merged with a miRNA normalized dataset to obtain a complete miRNA data table. Differential expression and statistical evaluation were determined using the “limma” library based on the linear model for microarray data [96]. *p*-values were calculated using an empirical Bayes moderated *t*-test with false discovery rate (FDR) correction for multiple tests. The selection criteria for significantly regulated miRNA expression were based on the difference between folds greater than absolute five and *p*-value after FDR correction (adj.p.val) < 0.05. Obtained results were visualized as scatter plots showing the total number of miRNAs up- and downregulated. Differentially expressed miRNA were also presented as heat maps and tables. Raw data files were deposited in the Gene Expression Omnibus (GEO) repository at the National Center for Biotechnology Information (http://www.ncbi.nlm.nih.gov/geo/, accessed on 2 December 2021) under the GEO accession number GEO: GSE190245. Public on 2 December 2021.

### 4.6. miRNA-Target Gene Prediction 

To identify potential target genes for differently expressed miRNA, we applied the SpidermiR library, where symbols of differentially expressed miRNAs were used to find target genes in the following databases: for predicted targets—DIANA, Miranda, PicTar, TargetScan, and for experimentally validated targets—miRTAR, miRwalk [97]. The mRNA transcriptomic data from our published experiment were used to determine the actual expression value of the target genes [40,41,42]. Obtained fold change values for mRNA were merged with the target genes data table. We selected for further analysis only target genes whose fold change was inversely correlated with the fold change value of appropriate miRNA (cut-off criteria: fold ±5, adjusted *p* value (adj.p.val.) <0.05). From the entire collection of miRNA–targets pairs, we selected only those that were involved in drug resistance, extracellular matrix, and cancer stem cell biology applying the following GO terms keywords: “collagen-containing extracellular matrix”, “extracellular matrix”, “extracellular space”, “response to drug”, and “stem cell”. Interactions between miRNA and target genes in relation to selected GO terms were visualized using Cytoscape 3.7.2 (National Institute of General Medical Sciences, Bethesda, MD, USA) [98].

## 5. Conclusions

In conclusion, we identified miRNAs that may be specific to CIS or PAC resistance, as their expression changes were observed in both CIS- and PAC-resistant cell lines. Some of them were previously described in the drug resistance or ovarian cancer progression, while the expression of others was observed for the first time. We also noted an inverse correlation between the expression of miRNAs and their targets, for a few pairs observed for the first time. The relation between new pairs needs to be confirmed at a molecular level. The significance of this regulation must be explained in the context of the resistance to cytotoxic drugs.

## Figures and Tables

**Table 1:** List of the miRNA fold changes, and false discovery rate (FDR) corrected *p* values (adj.p.val.). Each comparison was performed in relation to A2780 cells. miRs changes in two cell lines resistant to the same drug or changes over 20-fold are bolded.

Accession Number	Gene Name	A2780CR1	Adj P. Val A2780CR1	A2780CR2	Adj P. Val A2780CR2	A2780PR1	Adj P. Val A2780PR1	A2780PR2	Adj P. Val A2780PR2
MIMAT0000073	hsa-miR-19a-3p	−1.65917506	0.43228235	−1.98693607	0.35808508	−2.98536661	0.03505245	−10.4632712	5.01 × 10^−5^
MIMAT0000253	hsa-miR-10a-5p	1.11097524	0.92323367	1.16320532	0.93585887	**−42.4402572**	3.27 × 10^−7^	−1.08606811	0.97947299
MIMAT0000254	hsa-miR-10b-5p	−1.45872281	0.55603288	1.15064247	0.93234778	**−31.3998589**	6.48 × 10^−8^	1.05296136	0.98398973
MIMAT0004556	hsa-miR-10b-3p	−1.29212503	0.67767455	−2.01786429	0.21974679	**−39.3922097**	5.00 × 10^−9^	−1.70229117	0.44748
MIMAT0000423	hsa-miR-125b-5p	**11.4211844**	2.92 × 10^−5^	**40.6263024**	6.08E-08	1.20307908	0.81589843	11.7824642	2.23 × 10^−5^
MIMAT0004603	hsa-miR-125b-2-3p	4.51279562	0.00034004	12.4460487	6.7 × 10^−7^	1.10312933	0.88418483	−1.65376194	0.46375643
MIMAT0003233	hsa-miR-551b-3p	−10.2371817	0.00016236	−2.42299533	0.26166015	**−8.30014759**	0.00035114	**−43.0163879**	3.37 × 10^−7^
MIMAT0004794	hsa-miR-551b-5p	−4.08033818	0.00021598	1.02804528	0.98248749	**−8.7655429**	9.75 × 10^−7^	**−14.4305295**	7.61 × 10^−8^
MIMAT0000097	hsa-miR-99a-5p	**6.51698573**	0.0002292	**15.9710369**	1.99 × 10^−6^	−1.64710083	0.3694977	−6.10435252	0.00058133
MIMAT0000098	hsa-miR-100-5p	3.59432852	0.0002292	7.54674629	7.84 × 10^−7^	−1.81342348	0.08262989	**27.0328457**	4.86 × 10^−10^
MIMAT0000255	hsa-miR-34a-5p	−1.37990069	0.69602546	2.64309288	0.19626401	17.6185741	7.54 × 10^−6^	−1.28683148	0.88713934
MIMAT0000266	hsa-miR-205-5p	1.14870533	0.82352945	1.28436927	0.77623732	**541.061402**	5.72 × 10^−14^	1.16823451	0.90550058
MIMAT0000269	hsa-miR-212-3p	−10.7291338	1.38 × 10^−7^	−3.28929656	0.00114186	−4.37436267	4.22 × 10^−5^	−1.65503374	0.29893193
MIMAT0000275	hsa-miR-218-5p	**−17.621278**	2.9 × 10^−11^	**−19.7140856**	1.1 × 10^−11^	**−23.6716147**	2.01 × 10^−12^	**−20.6489829**	1.04 × 10^−11^
MIMAT0000278	hsa-miR-221-3p	**−29.1177907**	3.61 × 10^−9^	**−6.46450917**	1.91 × 10^−5^	**7.0380716**	7.54 × 10^−6^	**5.93519303**	3.51 × 10^−5^
MIMAT0000279	hsa-miR-222-3p	**−25.6406319**	1.77 × 10^−9^	**−7.69102525**	1.18 × 10^−6^	**10.8507703**	1.22 x10^−7^	**6.98808889**	2.74 × 10^−6^
MIMAT0000435	hsa-miR-143-3p	1.10184923	0.92330223	3.61736322	0.04419363	**51.5911963**	5.58 × 10^−8^	−2.05065576	0.39919454
MIMAT0000437	hsa-miR-145-5p	1.56380456	0.5547751	5.19999848	0.00954999	**33.1841961**	5.35 × 10^−7^	1.13346619	0.9609715
MIMAT0000446	hsa-miR-127-3p	1.27885145	0.72151884	**22.0754848**	2.01 × 10^−7^	6.77019849	0.00010088	−1.0050234	0.99757829
MIMAT0000447	hsa-miR-134-5p	1.73066708	0.35462069	19.4393236	8.85 × 10^−7^	4.30931787	0.00288523	1.04986309	0.9857338
MIMAT0000617	hsa-miR-200c-3p	2.89984641	0.04105261	2.79275829	0.11093757	**43.7996754**	5.58 × 10^−8^	1.42295677	0.78103834
MIMAT0000722	hsa-miR-370-3p	1.1993129	0.73879999	10.0811135	3.68 × 10^−7^	3.63111596	0.00043284	−1.05947357	0.97809363
MIMAT0000733	hsa-miR-379-5p	1.3156419	0.64154083	**35.5350551**	6.58 × 10^−9^	9.29366996	5.39 × 10^−6^	−1.01200703	0.99713672
MIMAT0000737	hsa-miR-382-5p	1.18956511	0.78650898	13.2070959	4.72 × 10^−7^	2.61591808	0.01777415	−1.15613669	0.917713
MIMAT0000738	hsa-miR-383-5p	1.61130832	0.51993375	3.39502246	0.07962031	**−23.308018**	2.64 × 10^−6^	3.7157868	0.04800945
MIMAT0001639	hsa-miR-409-3p	1.15628694	0.8803879	**37.5070461**	2.25 × 10^−7^	3.45870003	0.02255914	−1.07869628	0.97947299
MIMAT0002814	hsa-miR-432-5p	1.10745275	0.90787634	**30.3020757**	8.97 × 10^−8^	2.98721279	0.02300484	−1.00458053	0.99757829
MIMAT0002816	hsa-miR-494-3p	2.21477167	0.08016448	15.7339127	4.72 × 10^−7^	6.3051465	7.86 × 10^−5^	1.04777446	0.98403533
MIMAT0003161	hsa-miR-493-3p	1.40219291	0.56211501	13.7967448	9.3 × 10^−7^	3.73354575	0.00254391	1.02393083	0.99330976
MIMAT0003180	hsa-miR-487b-3p	−1.11202038	0.89278332	**24.6411571**	6.08 × 10^−8^	4.91920683	0.00041598	−1.11767275	0.95525848
MIMAT0004679	hsa-miR-296-3p	**25.7602551**	3.61 × 10^−9^	**26.0457953**	3.68 × 10^−9^	5.52181059	2.73 x10^−5^	−1.72053781	0.32752008
MIMAT0004951	hsa-miR-887-3p	**8.39589708**	4.6 × 10^−5^	**11.275164**	7.4 × 10^−6^	1.00761536	0.99337741	30.7206627	7.61 × 10^−8^
MIMAT0015052	hsa-miR-3175	19.7942794	0.04723357	14.0706299	0.17649319	2.31852234	0.67723594	3.4737797	0.6900128
MIMAT0015079	hsa-miR-3195	10.4085367	2.4 × 10^−8^	2.47389546	0.00689315	9.66553716	3.15 × 10^−8^	3.82713961	5.01 × 10^−5^
MIMAT0018085	hsa-miR-3663-3p	5.92541055	0.00078701	1.63225249	0.60726441	15.958959	5.39 × 10^−6^	1.73712026	0.57629842
MIMAT0018198	hsa-miR-3923	1.06673682	0.81082019	17.4769566	6.59 × 10^−13^	−1.37200803	0.06414619	−1.16479559	0.64994842
MIMAT0018929	hsa-miR-4417	11.8440191	0.02326632	1.30060439	0.94186674	1.86127822	0.67559363	3.59369405	0.49679601
MIMAT0018968	hsa-miR-4449	11.5059173	0.00042964	4.67501587	0.05941838	14.4465126	0.00016476	3.43350257	0.16510969
MIMAT0019019	hsa-miR-4485	7.6322304	0.06607829	1.84506285	0.81936494	**13.5381777**	0.0140913	**9.45065718**	0.08956618
MIMAT0019071	hsa-miR-4532	10.4128366	7.15 × 10^−5^	1.90699341	0.43120819	5.25328845	0.00186925	1.69206066	0.61415103
MIMAT0019835	hsa-miR-4721	10.42426	0.00841055	4.74531569	0.19626401	4.16202262	0.13162343	1.64195812	0.84365422
MIMAT0023693	hsa-miR-6068	9.29895604	0.00222206	1.55260856	0.80558365	10.1836996	0.00152316	−1.45736725	0.85496978
MIMAT0023700	hsa-miR-6075	9.5994209	1.68 × 10^−5^	2.53750857	0.092133	10.6369796	5.11 × 10^−6^	1.34386032	0.78827722
MIMAT0023705	hsa-miR-6080	10.7480047	0.00022974	2.04085944	0.46555248	7.78354196	0.00098186	−1.24777312	0.91729339
MIMAT0028121	hsa-miR-7112-5p	12.603672	6.0 × 10^−5^	2.60552327	0.21041134	7.69985587	0.00042421	1.71625463	0.63266738
MIMAT0030991	hsa-miR-8064	4.40065316	0.00053009	1.05752722	0.96575158	14.2050075	4.10 × 10^−7^	−1.18934073	0.90308138

## Data Availability

Not applicable.

## Supplementary Materials

The following supporting information can be downloaded at: https://www.mdpi.com/article/10.3390/ijms23010526/s1.
