# Supplementary material for: The Profile of MicroRNA Expression and Potential Role in the Regulation of Drug-Resistant Genes in Cisplatin- and Paclitaxel-Resistant Ovarian Cancer Cell Lines
Source: Int J Mol Sci. 2022 Jan 4;23(1):526. doi: 10.3390/ijms23010526 (PMC8745655; doi:10.3390/ijms23010526)
Supplement: Supplementary file 1 [file ijms-23-00526-s001.zip › ijms-1516966-SI.pdf]

Table S1. List of the miRNA fold changes, and false discovery rate (FDR) corrected p values (adj.p.val.). Each comparison was performed in relation to A2780 cells.

Accession Number	Gene Name	A2780CR1	Adj.P.Val.C1_Control	A2780CR2	Adj.P.Val.C2_Control	A2780PR1	Adj.P.Val.P1_Control	A2780PR2	Adj.P.Val.P2_Control
MIMAT0000064	hsa-let-7c-5p	1.850517625	0.312353131	5.368658839	0.003447674	1.019327755	0.985719908	-1.414337019	0.784920269
MIMAT0000071	hsa-miR-17-3p	-1.826321678	0.119473773	-1.863781299	0.198732859	-2.746912405	0.004671896	-6.499786746	8.62E-06
MIMAT0000073	hsa-miR-19a-3p	-1.659175061	0.43228235	-1.986936066	0.358085082	-2.985366611	0.035052448	-10.46327121	5.01E-05
MIMAT0000076	hsa-miR-21-5p	-3.177745387	0.000522679	-2.674319999	0.007628601	1.459633072	0.29927345	-9.038784719	2.27E-07
MIMAT0004494	hsa-miR-21-3p	-1.647450054	0.517855019	-2.757033359	0.199648436	1.461303912	0.625773695	-8.926590589	0.000581333
MIMAT0000253	hsa-miR-10a-5p	1.110975243	0.92323367	1.16320532	0.935858866	-42.44025721	3.27E-07	-1.086068113	0.979472989
MIMAT0000254	hsa-miR-10b-5p	-1.458722813	0.556032878	1.150642474	0.932347783	-31.3998589	6.48E-08	1.052961361	0.983989728
MIMAT0004556	hsa-miR-10b-3p	-1.292125033	0.677674553	-2.017864286	0.219746791	-39.3922097	5.00E-09	-1.702291172	0.447480003
MIMAT0000423	hsa-miR-125b-5p	11.42118444	2.92E-05	40.62630242	6.08E-08	1.203079084	0.81589843	11.78246419	2.23E-05
MIMAT0004603	hsa-miR-125b-2-3p	4.512795622	0.000340036	12.44604873	6.74E-07	1.103129329	0.884184827	-1.653761936	0.463756428
MIMAT0003233	hsa-miR-551b-3p	-10.23718175	0.000162357	-2.422995328	0.26166015	-8.300147591	0.000351144	-43.01638785	3.37E-07
MIMAT0004794	hsa-miR-551b-5p	-4.08033818	0.000215976	1.028045279	0.982487492	-8.765542903	9.75E-07	-14.43052946	7.61E-08
MIMAT0000226	hsa-miR-196a-5p	-3.244464909	0.00115471	-2.047557884	0.112021986	-6.505051227	6.62E-06	-1.823380356	0.228079803
MIMAT0001080	hsa-miR-196b-5p	-3.744395037	0.043404572	-1.306628267	0.893655064	-7.355455364	0.00192112	-2.102567273	0.509544253
MIMAT0000097	hsa-miR-99a-5p	6.516985726	0.000229198	15.97103692	1.99E-06	-1.64710083	0.369497697	-6.10435252	0.000581333
MIMAT0000098	hsa-miR-100-5p	3.594328523	0.000229198	7.546746287	7.84E-07	-1.813423484	0.082629891	27.03284571	4.86E-10
MIMAT0000102	hsa-miR-105-5p	-1.425733494	0.400824724	-1.33452033	0.669553439	9.533964444	2.36E-07	-1.203406879	0.84928756
MIMAT0000242	hsa-miR-129-5p	2.377853356	0.022951662	1.094643387	0.941866739	5.684840194	3.18E-05	1.263211964	0.813233251
MIMAT0000255	hsa-miR-34a-5p	-1.379900692	0.696025458	2.643092881	0.196264005	17.61857407	7.54E-06	-1.286831483	0.887139337
MIMAT0000266	hsa-miR-205-5p	1.14870533	0.823529449	1.284369268	0.776237316	541.0614023	5.72E-14	1.168234506	0.905500583
MIMAT0000269	hsa-miR-212-3p	-10.72913375	1.38E-07	-3.289296561	0.001141859	-4.374362674	4.22E-05	-1.655033744	0.298931934
MIMAT0000275	hsa-miR-218-5p	-17.62127796	2.99E-11	-19.71408564	1.17E-11	-23.67161471	2.01E-12	-20.64898286	1.04E-11
MIMAT0000278	hsa-miR-221-3p	-29.1177907	3.61E-09	-6.464509174	1.91E-05	7.038071597	7.54E-06	5.935193031	3.51E-05
MIMAT0000279	hsa-miR-222-3p	-25.64063185	1.77E-09	-7.691025253	1.18E-06	10.8507703	1.22E-07	6.988088892	2.74E-06
MIMAT0000426	hsa-miR-132-3p	-5.194242211	6.02E-05	-2.325806365	0.059081327	-4.076359723	0.000248053	-1.016118328	0.994090271
MIMAT0000430	hsa-miR-138-5p	3.218596302	0.024223535	7.55814158	0.000419109	6.120793914	0.000579589	-1.365344687	0.817844186
MIMAT0000432	hsa-miR-141-3p	-1.030537716	0.984207058	1.019912834	0.995308067	9.545745301	0.000582007	-1.020268989	0.997005027

MIMAT0000435	hsa-miR-143-3p	1.101849231	0.923302233	3.617363218	0.044193634	51.59119635	5.58E-08	-2.050655759	0.399194544
MIMAT0000437	hsa-miR-145-5p	1.563804561	0.554775096	5.199998485	0.009549994	33.18419614	5.35E-07	1.133466194	0.960971504
MIMAT0000438	hsa-miR-152-3p	2.837618615	0.038375096	1.267827236	0.865365315	7.54457841	0.000120886	2.604354188	0.1326724
MIMAT0000446	hsa-miR-127-3p	1.278851452	0.721518843	22.07548475	2.01E-07	6.770198486	0.000100883	-1.005023396	0.997578291
MIMAT0000447	hsa-miR-134-5p	1.730667082	0.354620685	19.43932362	8.85E-07	4.309317868	0.002885226	1.049863087	0.985733796
MIMAT0000450	hsa-miR-149-5p	1.923156883	0.307244923	1.832332477	0.489401628	3.086776933	0.038740193	6.679674172	0.001153235
MIMAT0000617	hsa-miR-200c-3p	2.899846406	0.041052611	2.792758292	0.110937572	43.79967537	5.58E-08	1.422956769	0.78103834
MIMAT0000722	hsa-miR-370-3p	1.199312899	0.738799994	10.08111352	3.68E-07	3.631115963	0.000432843	-1.05947357	0.978093629
MIMAT0000733	hsa-miR-379-5p	1.3156419	0.641540834	35.5350551	6.58E-09	9.293669965	5.39E-06	-1.012007035	0.997136717
MIMAT0000737	hsa-miR-382-5p	1.189565105	0.786508976	13.20709593	4.72E-07	2.61591808	0.017774151	-1.156136692	0.917712998
MIMAT0000738	hsa-miR-383-5p	1.61130832	0.519933753	3.395022461	0.079620313	-23.30801801	2.64E-06	3.715786801	0.048009452
MIMAT0000764	hsa-miR-339-5p	-1.281471551	0.594348224	1.114335334	0.931696105	1.540927065	0.25537543	7.00925743	2.54E-06
MIMAT0001412	hsa-miR-18b-5p	-2.722425448	0.003944909	-1.834131771	0.196264005	-2.445637201	0.009628789	-7.029666916	2.92E-06
MIMAT0001625	hsa-miR-431-5p	1.000832327	0.999285573	7.528619644	1.21E-06	3.447546509	0.000421993	1.113562492	0.935258949
MIMAT0001639	hsa-miR-409-3p	1.156286943	0.880387901	37.50704613	2.25E-07	3.458700034	0.022559144	-1.078696275	0.979472989
MIMAT0002814	hsa-miR-432-5p	1.107452751	0.907876337	30.30207575	8.97E-08	2.987212789	0.023004836	-1.00458053	0.997578291
MIMAT0002816	hsa-miR-494-3p	2.214771666	0.080164475	15.73391267	4.72E-07	6.3051465	7.86E-05	1.047774461	0.984035328
MIMAT0003161	hsa-miR-493-3p	1.402192913	0.56211501	13.79674481	9.32E-07	3.733545745	0.002543906	1.023930832	0.993309756
MIMAT0003180	hsa-miR-487b-3p	-1.112020383	0.892783319	24.64115705	6.08E-08	4.919206834	0.000415977	-1.117672751	0.955258477
MIMAT0003237	hsa-miR-572	7.519963456	0.00112016	1.515540552	0.761253449	9.737352219	0.000327145	1.478536459	0.803438057
MIMAT0003297	hsa-miR-628-3p	1.462259774	0.7104739	7.040355653	0.012744382	1.67876104	0.556649626	-1.144002842	0.969878793
MIMAT0003882	hsa-miR-767-5p	-1.622929754	0.30022888	-1.770113193	0.305680158	6.104636876	3.38E-05	-1.684214313	0.412750762
MIMAT0004284	hsa-miR-675-5p	2.846705843	0.001635616	7.335497046	9.01E-07	4.282177725	5.16E-05	-1.0135421	0.994962765
MIMAT0004560	hsa-miR-183-3p	-1.240388504	0.723668517	1.579499317	0.48694691	-1.558804871	0.333251353	8.159995121	9.49E-06
MIMAT0004679	hsa-miR-296-3p	25.76025506	3.61E-09	26.04579526	3.68E-09	5.521810586	2.73E-05	-1.720537813	0.327520077
MIMAT0004702	hsa-miR-339-3p	1.10821808	0.773626668	-1.385899759	0.311037771	-1.010658567	0.981592928	5.006528848	1.54E-07
MIMAT0004703	hsa-miR-335-3p	-9.318580243	0.000105739	-3.531134336	0.044193634	-2.695782629	0.065290158	-1.95588381	0.435354077
MIMAT0004770	hsa-miR-516a-5p	1.185110781	0.788742622	1.212334883	0.865365315	7.686775016	8.85E-06	1.291275887	0.804327294
MIMAT0004819	hsa-miR-671-3p	6.518002825	0.000197888	2.223854288	0.214075334	-1.07136631	0.934403778	5.824609802	0.000635378
MIMAT0004948	hsa-miR-885-3p	8.738708636	9.67E-05	2.514960825	0.164019009	5.285493676	0.001102175	2.479633862	0.176629289
MIMAT0004951	hsa-miR-887-3p	8.395897085	4.68E-05	11.27516397	7.45E-06	1.007615365	0.993377414	30.72066269	7.61E-08
MIMAT0004982	hsa-miR-939-5p	5.264027136	0.000656521	-1.062223818	0.967563182	5.856353734	0.0003393	1.24471294	0.885756967
MIMAT0005572	hsa-miR-1225-5p	5.184595172	5.15E-05	1.502877952	0.485907928	3.753782372	0.000378085	1.048836595	0.979472989

MIMAT0005586	hsa-miR-1231	6.676877106	1.07E-06	2.082768494	0.038089513	4.55708988	8.54E-06	1.254151493	0.737667176
MIMAT0005865	hsa-miR-1202	13.44960929	9.18E-06	1.843747907	0.391484213	9.042824857	3.18E-05	2.11790832	0.28085633
MIMAT0007884	hsa-miR-1910-5p	5.506468797	5.15E-05	2.050498151	0.133191738	6.183642297	1.57E-05	2.913517207	0.009275828
MIMAT0014986	hsa-miR-3124-5p	5.807432233	0.00465308	1.309513656	0.887347322	4.515675933	0.014987725	1.066679157	0.985733796
MIMAT0015010	hsa-miR-3141	5.012677722	0.000437195	1.306782349	0.808411317	2.979787475	0.0137529	1.448054944	0.698908073
MIMAT0015052	hsa-miR-3175	19.79427944	0.047233566	14.07062994	0.176493186	2.318522343	0.677235935	3.473779701	0.690012803
MIMAT0015058	hsa-miR-3180-3p	5.278267716	1.66E-05	1.520028408	0.386139964	3.378045853	0.000302574	1.489230686	0.469232039
MIMAT0015061	hsa-miR-3181	3.310435983	0.001799716	1.207099826	0.859340056	7.796407237	4.64E-06	1.048006251	0.981328546
MIMAT0015065	hsa-miR-3185	6.68957557	9.94E-06	1.894877825	0.171809334	5.631968224	1.44E-05	1.389150675	0.642419513
MIMAT0015069	hsa-miR-3187-3p	3.806452189	0.072908619	-1.002744566	0.998473006	5.509542967	0.016452214	-2.46312183	0.446798603
MIMAT0015070	hsa-miR-3188	8.983792225	0.000390137	1.322571433	0.870383266	9.02700043	0.000367916	1.195813692	0.942484798
MIMAT0015079	hsa-miR-3195	10.40853667	2.48E-08	2.47389546	0.006893153	9.665537162	3.15E-08	3.827139611	5.01E-05
MIMAT0015082	hsa-miR-3197	5.648857904	0.003008205	1.360068252	0.8445286	7.881746564	0.000531967	1.487619651	0.783454307
MIMAT0015378	hsa-miR-3065-3p	4.71867433	0.000152173	5.653229024	4.88E-05	3.969737486	0.000382096	-1.244533283	0.831650669
MIMAT0016873	hsa-miR-4322	5.073082507	0.000283004	1.229076911	0.865365315	4.131219678	0.001106062	-1.168667375	0.917293392
MIMAT0017991	hsa-miR-3613-3p	1.158582612	0.879452786	-1.792501857	0.52130161	-5.398850691	0.002080166	1.826068039	0.547490848
MIMAT0018068	hsa-miR-3648	5.846671364	0.002953318	1.15322489	0.941866739	5.517155475	0.003826645	1.34172558	0.865090072
MIMAT0018085	hsa-miR-3663-3p	5.925410546	0.000787008	1.632252489	0.607264414	15.95895903	5.39E-06	1.737120257	0.57629842
MIMAT0018115	hsa-miR-3687	5.928525539	0.018235742	1.159809901	0.954895754	1.36965121	0.781634789	2.540263248	0.463508734
MIMAT0018178	hsa-miR-3180	5.117297705	6.29E-05	1.448917527	0.560495566	3.330136165	0.001144631	1.479168903	0.570188944
MIMAT0018198	hsa-miR-3923	1.066736816	0.810820191	17.47695663	6.59E-13	-1.372008032	0.064146193	-1.164795592	0.649948423
MIMAT0018352	hsa-miR-3937	7.292307172	1.68E-05	1.882011825	0.228940763	6.225926699	2.40E-05	2.599879662	0.033774927
MIMAT0018444	hsa-miR-642b-3p	2.655896589	0.047386914	1.957929156	0.326715696	5.364944393	0.000608677	-1.429961748	0.749881163
MIMAT0018929	hsa-miR-4417	11.84401913	0.02326632	1.300604391	0.941866739	1.861278221	0.675593634	3.593694051	0.496796013
MIMAT0018968	hsa-miR-4449	11.5059173	0.000429638	4.675015868	0.059418376	14.44651259	0.000164755	3.433502569	0.165109686
MIMAT0019019	hsa-miR-4485	7.632230397	0.066078294	1.84506285	0.819364944	13.5381777	0.014091302	9.450657177	0.089566181
MIMAT0019032	hsa-miR-4497	5.268468802	0.000128999	1.431561731	0.632685669	3.470331924	0.001841535	1.18745471	0.895622318
MIMAT0019033	hsa-miR-4498	5.761182671	6.78E-05	1.425278129	0.636644764	3.842029594	0.000788948	1.239507354	0.84928756
MIMAT0019041	hsa-miR-4505	5.003646319	0.001255266	1.691557503	0.527581385	3.955125782	0.005172618	1.795364733	0.496796013
MIMAT0019071	hsa-miR-4532	10.4128366	7.15E-05	1.906993412	0.43120819	5.253288448	0.001869247	1.692060656	0.61415103
MIMAT0019077	hsa-miR-1587	5.401911977	0.002202176	1.616769166	0.651184714	4.059299797	0.010218052	1.765296618	0.584947404
MIMAT0019082	hsa-miR-4539	3.090058994	0.002017061	1.542881809	0.445742022	6.540104913	7.54E-06	1.266626384	0.798405917
MIMAT0019691	hsa-miR-4634	9.828249401	4.52E-06	2.047145497	0.176052515	9.92545213	1.83E-06	2.057881895	0.172430538
MIMAT0019699	hsa-miR-4640-5p	5.215306838	0.000148124	1.184579432	0.892434324	5.352658392	9.23E-05	2.169022021	0.131975918
MIMAT0019721	hsa-miR-4655-5p	4.230633819	5.72E-05	1.100015677	0.932347783	5.132216766	7.82E-06	1.718737702	0.221766639
MIMAT0019723	hsa-miR-4656	6.105292112	6.07E-05	2.46639398	0.068951543	5.246054899	0.000116362	1.925070065	0.253824885
MIMAT0019739	hsa-miR-4665-5p	5.664571107	1.50E-05	1.110257487	0.931910636	5.249309411	1.27E-05	1.652308239	0.317342968
MIMAT0019778	hsa-miR-4689	6.419417842	0.005171613	1.545045889	0.779878645	5.31154235	0.011948951	1.381280166	0.872314128
MIMAT0019779	hsa-miR-4690-5p	5.567079904	1.68E-05	1.825456431	0.181293235	3.784675522	0.000170392	1.592932376	0.377265408

MIMAT0019788	hsa-miR-4695-5p	5.298276059	0.000107322	1.538942123	0.504336493	4.120229298	0.000430244	1.119915044	0.946182194
MIMAT0019807	hsa-miR-4707-5p	5.259973225	3.06E-05	1.311360846	0.712969873	4.499145698	6.60E-05	1.327282864	0.711381586
MIMAT0019835	hsa-miR-4721	10.42426002	0.008410552	4.745315693	0.196264005	4.16202262	0.131623426	1.641958121	0.843654224
MIMAT0019844	hsa-miR-4725-3p	4.471596342	0.001933137	-1.249560785	0.872161451	7.029540569	0.000129409	-1.220242688	0.903840006
MIMAT0019871	hsa-miR-4741	5.394450171	1.28E-05	1.601421022	0.293306045	5.884274696	2.99E-06	1.11393307	0.926570026
MIMAT0019887	hsa-miR-4750-5p	9.932022592	0.000107917	1.839968614	0.485572537	5.463903585	0.001767644	2.081379468	0.390003771
MIMAT0019913	hsa-miR-4763-3p	5.169087576	0.000145028	1.439391815	0.624528746	4.196231243	0.000432804	1.350121994	0.74055151
MIMAT0019919	hsa-miR-4767	3.043705726	0.02923395	1.146224253	0.935858866	5.823023058	0.000634748	1.429148657	0.773174866
MIMAT0019947	hsa-miR-4783-3p	4.797928338	0.000263353	1.995407199	0.214075334	7.177256265	1.96E-05	-1.052884933	0.980122436
MIMAT0019979	hsa-miR-4800-3p	8.424071275	9.31E-05	1.669082128	0.54553594	7.560220503	0.000118239	1.878374177	0.438445007
MIMAT0021120	hsa-miR-5189-5p	7.028074856	1.84E-05	2.567103004	0.040809821	6.84314622	1.16E-05	1.577785042	0.497927491
MIMAT0022259	hsa-miR-5100	5.225760661	0.005047593	1.975296035	0.469642947	1.864742407	0.356678141	2.82914761	0.191430598
MIMAT0022260	hsa-miR-5572	6.848611005	0.00023509	-1.003628447	0.99803823	3.985384299	0.005320416	1.05967376	0.982343111
MIMAT0022938	hsa-miR-937-5p	5.649071218	0.000291973	1.665451132	0.497281606	4.559865438	0.001108863	1.407106556	0.750488052
MIMAT0022942	hsa-miR-1229-5p	6.086426057	0.01384524	-1.720161701	0.730797713	3.57104139	0.095567862	1.138493354	0.975930024
MIMAT0022977	hsa-miR-4632-5p	5.052931067	0.000523857	1.456361253	0.678312126	4.015915549	0.002284953	1.499206862	0.667257418
MIMAT0023693	hsa-miR-6068	9.298956041	0.002222064	1.552608562	0.805583652	10.18369957	0.001523155	-1.457367254	0.854969781
MIMAT0023700	hsa-miR-6075	9.599420897	1.68E-05	2.537508571	0.092133	10.63697963	5.11E-06	1.343860322	0.788277218
MIMAT0023705	hsa-miR-6080	10.74800465	0.000229737	2.040859436	0.465552483	7.783541962	0.000981863	-1.247773119	0.917293392
MIMAT0024599	hsa-miR-6126	5.644287504	0.009098972	1.40206218	0.85083269	4.092237626	0.035130183	1.312557599	0.905500583
MIMAT0024616	hsa-miR-6132	6.653745068	0.008607039	1.675233278	0.739164366	5.333746687	0.020501237	1.978687309	0.637226541
MIMAT0025476	hsa-miR-6510-5p	5.3481113	0.000983733	1.584199418	0.624846673	3.36380922	0.015121514	1.63323045	0.620524061
MIMAT0025844	hsa-miR-6716-5p	5.80465124	0.007075513	1.091820698	0.965751578	3.724327677	0.048062509	1.46726898	0.82943564
MIMAT0025855	hsa-miR-6723-5p	3.533088352	0.002998113	1.722736966	0.389620126	6.12131924	7.68E-05	1.476470774	0.649948423
MIMAT0027375	hsa-miR-6737-5p	5.484286911	1.68E-05	1.206516571	0.823450003	6.812742122	1.89E-06	1.743236766	0.232222144
MIMAT0027406	hsa-miR-6753-5p	5.043875589	0.000207423	1.036649935	0.978437335	4.89118725	0.000238319	1.084620485	0.969572457
MIMAT0027426	hsa-miR-6763-5p	6.558848866	0.000277577	1.767991889	0.472632711	4.63856373	0.002090956	1.400761315	0.784920269
MIMAT0027436	hsa-miR-6768-5p	4.394368305	0.001350025	-1.432476566	0.706890443	6.979407547	8.06E-05	-1.683111202	0.525192932
MIMAT0027458	hsa-miR-6779-5p	5.172426219	6.29E-05	1.620381284	0.372849974	4.455487152	0.000128402	2.364253513	0.048009452
MIMAT0027464	hsa-miR-6782-5p	5.273766247	0.000279899	-1.016466441	0.995308067	2.469538549	0.042256663	1.699250949	0.482230645
MIMAT0027474	hsa-miR-6787-5p	5.322982879	9.41E-05	1.448650256	0.597197881	3.230528956	0.002522176	1.914211349	0.228079803
MIMAT0027480	hsa-miR-6790-5p	9.175840509	0.000690048	1.684040156	0.686391118	7.730564259	0.001523155	1.229817111	0.931608311
MIMAT0027496	hsa-miR-6798-5p	6.045089858	1.48E-05	1.575000774	0.375678312	4.29860196	9.19E-05	1.79866887	0.224524432
MIMAT0027498	hsa-miR-6799-5p	6.033168249	0.00115471	1.032725944	0.989649685	5.630099664	0.001724197	-1.108170021	0.972545722
MIMAT0027500	hsa-miR-6800-5p	4.609755674	0.0017745	1.334273997	0.813466762	5.051549243	0.000994348	1.211794046	0.91215864
MIMAT0027504	hsa-miR-6802-5p	7.781125542	0.000229198	1.640152577	0.612543234	5.69076238	0.001177294	1.996727277	0.419704764
MIMAT0027524	hsa-miR-6812-5p	5.89796255	3.15E-05	2.258310729	0.078473856	4.390765512	0.000169051	1.528324449	0.52517884
MIMAT0027540	hsa-miR-6820-5p	6.895768515	3.37E-05	1.966712072	0.220949681	5.747839292	7.34E-05	2.276692141	0.110988498

MIMAT0027583	hsa-miR-6840-3p	6.10430385	3.61E-09	-1.165995265	0.720752336	3.088420611	2.93E-06	-1.027642013	0.979472989
MIMAT0027590	hsa-miR-6845-5p	5.389825839	0.000265773	1.33334865	0.78052247	5.287064852	0.000297176	1.349719747	0.784920269
MIMAT0027622	hsa-miR-6860	7.680176265	2.46E-05	1.404642649	0.680195457	6.017660883	6.15E-05	1.314523488	0.79060221
MIMAT0027623	hsa-miR-6861-5p	5.700762896	0.000127512	1.509859386	0.57453491	5.260220365	0.000154993	1.152377864	0.925137926
MIMAT0027678	hsa-miR-6889-5p	4.629014592	0.004189522	1.037694694	0.986036642	8.809790721	0.000127101	1.368984437	0.829301015
MIMAT0028109	hsa-miR-7106-5p	5.387623732	0.001681284	1.225614818	0.910760772	4.982389468	0.00257189	1.053713478	0.985787081
MIMAT0028115	hsa-miR-7109-5p	8.279769744	0.000181108	1.77883254	0.514330952	3.854850761	0.010812282	3.758787572	0.031211928
MIMAT0028117	hsa-miR-7110-5p	4.414589207	0.010076164	4.424062522	0.028568824	6.212048996	0.001683284	1.858694029	0.568336154
MIMAT0028121	hsa-miR-7112-5p	12.60367197	6.02E-05	2.605523271	0.210411342	7.699855871	0.000424212	1.716254629	0.632667375
MIMAT0030420	hsa-miR-7845-5p	6.60651556	1.68E-05	2.083652904	0.116102235	7.713180747	3.27E-06	2.324135999	0.056332187
MIMAT0030990	hsa-miR-8063	6.740321988	0.007420195	1.198341797	0.937730682	7.84615519	0.003756771	-1.479942329	0.848435802
MIMAT0030991	hsa-miR-8064	4.400653161	0.00053009	1.057527217	0.965751578	14.20500752	4.10E-07	-1.189340728	0.903081382
MIMAT0031000	hsa-miR-8073	5.281423445	0.000246564	1.571927885	0.541396574	4.516793146	0.000638208	1.280531144	0.837803861
